# Are we under-utilizing the talents of primary care personnel? A job analytic examination

**DOI:** 10.1186/1748-5908-2-10

**Published:** 2007-03-30

**Authors:** Sylvia J Hysong, Richard G Best, Frank I Moore

**Affiliations:** 1 Houston Center for Quality of Care & Utilization Studies, Michael E. DeBakey VA Medical Center, Houston, TX, USA; 2 Department of Medicine, Baylor College of Medicine, Houston, TX USA; 3 Lockheed Martin Business Process Solutions, San Antonio, TX USA; 4 School of Public Health, University of Texas Health Science Center at Houston – San Antonio Regional Campus, San Antonio, TX USA

## Abstract

**Background:**

Primary care staffing decisions are often made unsystematically, potentially leading to increased costs, dissatisfaction, turnover, and reduced quality of care. This article aims to (1) catalogue the domain of primary care tasks, (2) explore the complexity associated with these tasks, and (3) examine how tasks performed by different job titles differ in function and complexity, using Functional Job Analysis to develop a new tool for making evidence-based staffing decisions.

**Methods:**

Seventy-seven primary care personnel from six US Department of Veterans Affairs (VA) Medical Centers, representing six job titles, participated in two-day focus groups to generate 243 unique task statements describing the content of VA primary care. Certified job analysts rated tasks on ten dimensions representing task complexity, skills, autonomy, and error consequence. Two hundred and twenty-four primary care personnel from the same clinics then completed a survey indicating whether they performed each task. Tasks were catalogued using an adaptation of an existing classification scheme; complexity differences were tested via analysis of variance.

**Results:**

*Objective one*: Task statements were categorized into four functions: service delivery (65%), administrative duties (15%), logistic support (9%), and workforce management (11%). *Objective two*: Consistent with expectations, 80% of tasks received ratings at or below the mid-scale value on all ten scales. *Objective three*: Service delivery and workforce management tasks received higher ratings on eight of ten scales (multiple functional complexity dimensions, autonomy, human error consequence) than administrative and logistic support tasks. Similarly, tasks performed by more highly trained job titles received higher ratings on six of ten scales than tasks performed by lower trained job titles. Contrary to expectations, the distribution of tasks across functions did not significantly vary by job title.

**Conclusion:**

Primary care personnel are not being utilized to the extent of their training; most personnel perform many tasks that could reasonably be performed by personnel with less training. Primary care clinics should use evidence-based information to optimize job-person fit, adjusting clinic staff mix and allocation of work across staff to enhance efficiency and effectiveness.

## Background

Health care systems spend up to as much as two-thirds of their non-capital budget on personnel [1,2], yet staffing decisions such as staff mix in primary care clinics or distribution of work among clinic personnel are often made unsystematically. Work is often assigned to whoever is available rather than whoever is best qualified for the task [3]. Decisions like these, without a suitable evidence base to support them, are counterproductive in two important ways. First, they may utilize more highly trained, more expensive personnel for administrative or simple tasks that could be performed by less expensive personnel. Second, they may require significant staff time for tasks below the full use of employees' skills and training. This can be detrimental to employee satisfaction. Recent work has noted that the rate of young physicians leaving internal medicine is significantly higher in primary care than in other subspecialties of internal medicine, with dissatisfaction with working conditions as one of several important reasons for leaving [4]. Increased turnover for similar reasons has also been noted among other primary care staff, both clinical and administrative [5]. Beyond increased turnover [6,7], clinician dissatisfaction has also been associated with increases in medical errors [8] and decreases in productivity [9,10] and quality of care [7,11]. Finally, staffing decisions directly affect quality of care: new models of care and evidence-based practice (e.g., chronic care model, clinical practice guidelines) often require changes in staffing levels, configurations [12], and coordination patterns [13] to produce successful, sustainable improvements.

Emulating initiatives to implement evidence-based practice interventions, researchers and policy makers now advocate evidence-based management in health care [14], promoting use of management practices with solid evidence of effectiveness while avoiding management practices with weak effectiveness evidence [15,16]. Just as implementing evidence-based practice interventions (e.g., incorporating a care coordinator or care manager into a clinic, introducing an electronic medical record, or implementing clinical practice guidelines) requires reliable, valid data or evidence, so too does the implementation of evidence-based management interventions. In primary care staffing, this requires detailed information about the nature of primary care work, and its requisite knowledge, skills, and abilities [17-19]. Reliable and valid information about the nature and requirements of work, the worker, and the work environment is obtained via a job analysis.

### Job analysis in health care settings

Formal job analysis techniques have been used for decades in most industries as the basis for important human resource decisions. Job analytic information is collected systematically from multiple sources (e.g., job incumbents, supervisors, archival information) via several methods (e.g., survey, interviews, direct observation) and is used for multiple purposes (e.g., determining ideal qualifications for new hires, identifying skill sets in which the current workforce might need training, establishing criteria for performance evaluation).

In health care, job analysis has been advocated as a useful tool for redesigning effective and efficient systems of care; various job analytic techniques have been used and advocated within healthcare for numerous applications [20]. Mbambo and colleagues used a task inventory to clarify job expectations and assess skill mix for different categories of nurses in a district health system in South Africa; they found that hospital nurses had higher job demands and lower job resources than other categories of nurses and were therefore more at risk of burnout, despite having many tasks in common with other types of nurse [21]. Task inventories have also been used to develop [22] and validate Occupational Health Nurse certification exams [23]. The validation study found that Certified Occupational Health Nurses (COHNs) were more likely to function as clinicians, whereas COHN-specialists were more likely to function as educators and managers, thus supporting the need for separate certification exams. Soh advocated the use of job analytic techniques as an essential prerequisite to assessing surgeon performance [24]. Dreesch and colleagues [25] recommended a methodology based on a service target approach and Functional Job Analysis (FJA, the technique used in this article) to estimate the human resource requirements for meeting the population health services delivery goals set forth by the United Nations Millenium Declaration. These projects all highlight the flexibility of job analytic techniques and their value as the foundational information source for making evidence-based staffing decisions.

### Reforming health care delivery: the Colombia Ministry of Health project

One of the most significant, extensive applications of job analysis in health care occurred in Colombia [26], where the Ministry of Health used FJA data as part of a major health care delivery system reform effort. Several important findings emerged from this work:

• Substantial overlap (over 80%) existed in tasks performed by physicians, nurses, and auxiliary nurses, signifying tremendous opportunity for more efficient distribution of primary care work among existing personnel.

• Primary care was a highly prescribed work environment with little opportunity to exercise independent judgment, often resulting in low satisfaction and higher than expected turnover.

• The complexity of primary care work rarely exceeded middle levels of difficulty, supporting the conclusion that assigning doctors and, to a lesser extent, professional nurses to the bulk of the tasks involved in primary care was not the best use of these scarce and expensive resources.

These and other findings were used by the Ministry of Health to create a system-wide task bank and redesign the various roles of primary care personnel, achieving cost savings of over 1.5 million pesos ($906 adjusted for inflation) per person per month (about twice the monthly salary of a staff nurse), and significant increases in employee satisfaction.

Despite the wide-reaching changes possible from the strategic use of a system-wide task bank such as the one developed for Colombia, such technology has gone largely unnoticed in American primary care. To our knowledge, job analytic (especially FJA) data have not been used for strategic human resource change involving an entire work system with multiple occupations such as primary care. This article is one of two papers reporting the results of a large study that examined staffing patterns in VA primary care via the use of a primary care task bank [27]. The current article documents the domain of work conducted in primary care, its complexity, and differences in complexity by function and occupation. The first article [28] documented the extent to which primary care tasks are performed by multiple occupations in a primary care clinics (suggesting potential redundancy of work and opportunity to improve efficiency) and illustrated how job analytic data can be used to perform work reallocation. The current article has three objectives:

1. Catalogue the domain of tasks that constitute primary care.

2. Characterize the complexity associated with these tasks.

3. Examine how tasks performed by different job titles differ in function and complexity.

In support of the latter two objectives we propose the following hypotheses, based on the Colombia Ministry of Health project's findings [29]:

Hypothesis 1. Primary care tasks will not exceed moderate levels of complexity.

Hypothesis 2. Complexity of primary care tasks will vary significantly by function (e.g., medical procedures will exhibit higher complexity ratings than clerical tasks).

Hypothesis 3. Complexity of primary care tasks will vary significantly by job title, such that tasks performed by higher trained personnel (e.g., MDs, advanced practitioners) will exhibit higher complexity ratings than tasks performed by personnel with less training (e.g., health technicians).

Hypothesis 4. Differences in complexity ratings of tasks performed by different job titles will depend on the content of tasks performed.

## Methods

### Site selection

We operated under the assumption that the work of primary care is invariant across VA facilities, but that the allocation of the work to specific job titles would vary by facility. Six VA medical centers participated in the study, based on the following criteria identified by an expert advisory panel as likely to influence staffing patterns and the work conducted in primary care: medical school affiliation (more likely to perform precepting tasks), size (smaller facilities are less likely to have specialty personnel in primary care, such as social workers or nutritionists), past history as primarily a psychiatric inpatient unit (likely to affect the amount of mental health work performed in primary care), and the implementation of advanced clinic access in primary care. Also known as open scheduling or same-day scheduling, Advanced Clinic Access refers to a process popularized by the Institute for Healthcare Improvement (IHI) to reduce appointment congestion, no-shows, and appointment wait times, and was deemed likely to affect workflow patterns. Of the available sites meeting these criteria, the final sites were selected on the basis of feasibility of scheduling and travel and the availability of participants. Table 1 lists how the six sites compare on these and other characteristics.

**Table 1:** Site characteristics and number of focus group participants by site

	Participating facility						
		
	1	2	3	4	5	6	Total
*Site characteristics*							
Advanced clinic access implementation	Y	Y	N	N	Y	N	
Inpatient/residential psychiatric facility	Y	N	N	Y	Y	N	
Academically affiliated	Y	N	Y	Y	Y	Y	
Number of employees	1006	859	2183	1211	2907	608	
Average patient commute (miles)	4.63	15.53	8.4	17.15	3.6	22.14	
							
*Number of focus group participants*							
Physician	--	--	4	5	--	5	14
PA/NP	6	--	6	--	--	6	18
RN	7	4	--	--	--	--	11
LVN	3	--	--	5	5	--	13
Clerk	--	3	--	--	6	--	9
Health technician	--	--	--	7	--	5	12

Total	16	7	10	17	11	16	77

### Participants

Seventy-seven primary care personnel from six primary care job titles (Physician, Nurse Practitioner/Physician Assistant, Registered Nurse, Licensed Vocational Nurse, Health Technician, and Clerk) across the six sites participated as subject matter experts (SMEs) in a total of 15 two-day focus groups. Separate focus groups were conducted for each job title (six to eight SMEs per focus group). The study's local principal investigator at each facility nominated suitable participant candidates, targeting incumbents with at least one year of experience and a record of high performance in their current position. To minimize facility burden, no more than three focus groups per site were conducted. To minimize any biases that may have ensued from the presence of a supervisor during the focus group, supervisory personnel (e.g., chief of staff of primary care) were excluded from the focus groups. Table 1 displays the distribution of job titles sampled at each facility and the number of SMEs participating in the focus groups. The same personnel who participated in focus groups also participated in a subsequent validation phase. For the verification (survey) phase, 224 out of a possible 619 employees across the six sites participated (36.19% response rate).

### Procedure

Various techniques exist for conducting a job analysis, including work-oriented methods such as task inventories and FJA [30,31], and worker-oriented methods such as skill-based surveys (e.g., the Position Analysis Questionnaire)[32] and the critical incidents technique [33]. For this study we employed FJA and its accompanying framework, Work-Doing Systems Theory [31]. Developed by Sidney Fine, this framework posits a dynamic interaction of three components of organizational systems: (1) the work organization (its purpose, goals, objectives); (2) the worker (capacities, experiences, education and training); and (3) the work content (the functions, sub-functions, activities, tasks and associated performance standards). FJA is the specific methodology used to describe the work content in the work-doing system.

FJA was particularly suited to accomplish our objective of developing a tool for making evidence-based staffing and work reallocation decisions for several reasons. First, recent research has shown that task-based job analytic techniques like FJA are more reliable and less biased than more generalized work activity techniques, such as competency modelling [34,35]. Second, worker-oriented techniques, whose chief purpose is to identify the dimensions required for performing a job well without detailing the tasks involved in performing the job, are inappropriate for work reallocation purposes because they do not capture the work content itself. Finally, FJA is a well-established methodology, with decades of research and use across many industries (including health care) to support it [30,31,36-40], as well as the technique with the widest range of applications due to the amount and variety of detail available for each task statement.

FJA methodology has been extensively documented elsewhere [31], and thus is only briefly explained here (Additional file 1 presents a brief primer). FJA uses task statements as the basic building blocks of human resource management and organizational strategic planning. Task statements explicitly incorporate the three components of work-doing systems using the following elements:

• Who (the worker)?

• Performs what action (work content)?

• With what tools, materials or work aids (work content)?

• Upon what instructions (including the requisite knowledge, skills, abilities, (worker characteristics) and performance standards for task performance)?

• To accomplish what organizational outcome or result (work organization)?

Tasks are also rated according to functional skill requirements that define the complexity of performance across cognitive, interpersonal, and physical dimensions, as well as potential consequence given an error in performance [41] (a brief description of the scales is provided in Table 2; see [31] for full descriptions). These ratings provide focus for what workers do in terms of the relative simplicity or complexity in their performance of the work content [40]; thus, the ratings provide additional guidance for decisions about task assignment. For example, tasks may be assigned to maximize the unique skills and expertise of workers (promoting employee growth and satisfaction), as well as to ensure competent personnel perform the work (enhancing quality of care and patient safety). Indeed, the rich array of information at the task level highlights the utility and flexibility in aligning the work with the requisite worker characteristics in service to the important organizational objectives. The advantage of this conceptualization is a more comprehensive architecture on which to examine current work patterns within the VA.

**Table 2:** Brief scale descriptions

*Things*: Physical interaction with and response to tangibles – touched, felt, observed, and related to in space; images visualized spatially.
*Data*: Interaction with information, ideas, facts, statistics, specification of output, knowledge of conditions, techniques; mental operations.
*People*: Live interaction among people, and between people and animals
*Worker Instructions*: The degree to which a task is completely prescribed by instructions to the worker, vs. left completely to the discretion of the worker.
*Reasoning Development*: Knowledge, ability to deal with theory versus practice, abstract versus concrete, and many versus few variables.
*Mathematical Development*: Knowledge and ability to deal with mathematical problems and operations from county and simple addition to higher mathematics.
*Language Development*: Knowledge and ability to speak, read, or write language materials from simple verbal instructions to complex sources or written information and ideas.
*Worker Technology*: Means and methods employed in completing a task or work assignment - tools, machines, equipment or work procedures, processes or any other aids to assist in the handling, processing or evaluation of things or data.
*Worker Interaction*: When working with others (through direct or indirect contact), workers assist them, coordinate their efforts with them and adapt their style and behavior to accommodate atypical or unusual circumstances and conditions. This effort results in achievement of employer goals to given standards.
*Human Error Consequence *– Degree of responsibility imposed upon the performer with respect to possible mental or physical harm to persons (including performer, recipients, respondents, co-workers, or the public) resulting from errors in performance of the task being scaled.

The present study used a modified FJA protocol composed of three phases: task generation, task validation, and task verification (traditional FJA only requires the first two). In task generation, FJA analysts facilitate focus groups with subject matter experts (SMEs, that is, incumbents of the job being analyzed) to co-create a list of task statements that describe the work performed by the incumbents. In task validation, the analysts edit the task statements for compliance with FJA syntax; SMEs and then review the task statements to ensure that they still accurately reflect the work they perform, and that at least 85% of the work they do is captured by the task statement list. Finally, because we were interested in the universe of tasks of a system of work (i.e., primary care), not simply a single job, a third step, task verification, was added to the process. In this step, incumbents reviewed their own task statements and the task statements of other primary care personnel to check for overlap and ensure no tasks had been missed.

#### Task generation

Two-day focus groups were conducted with the SMEs using a standard FJA focus group protocol [31] to generate tasks descriptive of their work. For each job title, task lists were generated de novo at the first site a given job title was encountered. For each subsequent site, SMEs reviewed the list of generated tasks, made edits as necessary, and generated any new tasks not already on the list.

#### Task validation

To ensure the reliability and validity of the task statements, three certified functional job analysts (all part of the research team) reviewed and edited the tasks to arrive at a consensus on the wording of each. To arrive at a consensus, each task was reviewed relative to nine criteria, such as whether the actions in the task statement logically result in the task statement's stated output, or whether performance criteria can be inferred from the language of the task statement. A full list of these criteria is presented in Additional file 1. Similar tasks that were generated by multiple focus groups were merged into a single task, to avoid redundancy in the task bank. SMEs then reviewed the edited tasks to ensure that they (a) accurately represented the work they did, (b) described the work clearly, and (c) captured at least 85% of the work performed by the job title in question. With the exception of the health technician tasks, which captured approximately 60% of the work they performed, all task banks met the above criteria. Health technicians were present in only two facilities, where they functioned in lieu of clerks but with the added responsibility of several clinical tasks not normally performed by clerks. Thus, we concentrated on their clinical tasks during their focus groups, which reduced the percent of work tasks captured by their focus group.

#### Task verification

The analysts rated the validated task statements along the ten work content dimensions prescribed by FJA: *data *(cognitive complexity), *people *(interpersonal complexity), *things *(physical/motor complexity), *reasoning, mathematics, language*, *worker instructions*, (autonomy), *worker technology *(complexity of methods employed in completing a task), *worker interaction *(complexity of interactions with other co-workers required to complete the task), and *human error consequence *(HEC, the seriousness of consequences resulting from completing the task incorrectly). The scales are briefly described in Table 2 and documented in detail elsewhere [40,42]. However, it is important to note that for the purposes of this paper, we use the term complexity to mean *the complexity of interactions with respect to the scale in question*. For example, a low data scale rating implies that the worker interacts with data in a very simple way, such as copying, as opposed to synthesizing data (the data itself can be complex – however if the *interaction *with the data is simple, then the task would receive a low rating on the scale).

A survey containing the finalized task bank across all job titles (n = 243) was distributed by the local principal investigator to all primary care personnel at each facility. Participants verified whether or not they performed each task (task endorsement), indicated how frequently they performed each task (frequency), and how long it took them to perform each task (duration).

## Results

### Preliminary analyses: cross-site comparisons

To test the assumption that primary care work was invariant across facilities, we compared the number of tasks shared by pairs of facilities. We found a high percentage of overlap among the tasks performed by any two sites (83%–95%), thus suggesting that primary care work is constant across facilities. To test the assumption that the distribution of work among primary care personnel varied across facilities, we calculated the number of sites endorsing a given task statement, grouped by job title (thus, a possible range of 0 – 6 for each task statement). Task statements that received values of 0 or 6 were considered invariant across the participating sites (i.e., all six sites agree that task x is or is not performed by job title y) whereas those receiving values of 1 – 5 were considered variant in the pattern of performance across sites (e.g., a value of "1" means that the task in question is performed by a given job title in one site, but not in the other 5). Task endorsement by each job title is significantly more variant than invariant across sites with the exception of LVNs (see Table 3), thus suggesting (consistent with our assumption) that responsibility in task performance varies across sites. Given these findings, we proceeded to perform analyses for each study objective.

**Table 3:** Chi-square goodness-of-fit test of concordant vs. discordant tasks across sites, by job title

	**Observed # of tasks**		
		
**Job title**	*Concordant*	*Discordant*	χ^2^*
Physician	83	160	25385.4
NP/PA	97	146	21111.8
RN	113	130	16709.7
LVN/LPN	139	104	19122.6
CLERK	108	135	11496.3
HT	84	159	25067.1

* All χ^2 ^values are significant at the .001 level, using both asymptotic and exact tests of significance. Expected N's for concordant and discordant tasks are 238 and 5, respectively.

Note: For job titles which did not exist at a particular facility, the concordance range was adjusted to fit the number of facilities for which that job title did exist.

### Objective one: cataloguing the domain of primary care tasks

**Figure 1:**
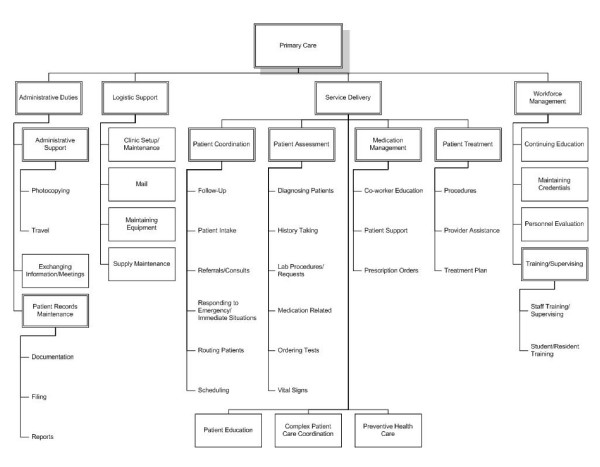
Hierarchical classification system of primary care work.

The finalized tasks were classified into a hierarchical system adapted from that used to describe public family planning clinics in the United States [40,43]. Inspired by Fine's early work [37], this system classifies tasks hierarchically by major function, sub-function, and activity rather than by structure (e.g., by occupation or organization unit). This is necessary because tasks are transportable across organization units and personnel classifications. It helps users to focus on what is done, rather than who is doing it and where in the organization it is happening. Though the functional structure was preserved, the primary care and family planning task banks differed sufficiently to warrant creating functions, sub-functions, and activities specific to the primary care task bank (e.g., activities such fundraising, developing of educational materials formed part of the family planning task bank but not the primary care task bank; similarly, sub-functions such as maintaining credentials and complex patient care coordination formed part of the primary care task bank, but not the family planning task bank). These categories emerged qualitatively from the tasks, [44] and are presented in Figure 1. The fully annotated task bank is available in electronic format [45].

Four major functions comprise the work of primary care:

1. **Service Delivery**. This function covers interactions between primary care personnel and the patient; most primary care tasks fall under this function (n = 158, or 65%). The service delivery function can be further divided into six sub-functions: patient assessment, patient treatment, patient coordination, preventive health care, patient education, and medication management. The largest sub-function is patient coordination (n = 60/158), which contains more tasks than patient assessment and patient treatment combined.

2. **Administrative Duties**. This function comprises the documentation and exchange of medical and non-medical information necessary for daily operations. This function can be further subdivided into three sub-functions: patient records maintenance, exchanging information in meetings, and administrative support (paperwork).

3. **Logistic Support**. This function covers the maintenance of primary care clinics, including supplies, equipment, and office space/examination rooms. This function can be subdivided into four sub-functions: clinic setup/maintenance, supply maintenance, maintaining equipment, and mail.

4. **Workforce Management**. This function is concerned with worker/worker relationships. Workforce management captures those actions dealing with the selection, training, direction, and evaluation of the workers in the facility. Many supervisory tasks fall within this function. The workforce management function can be subdivided into four sub-functions: training/supervising, continuing education, maintaining credentials, and personnel evaluation.

### Objective two: the complexity of FJA work content scales in primary care

**Figure 2:**
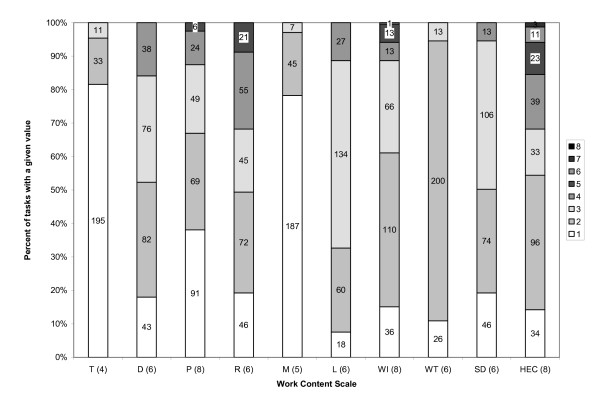
**Percent of tasks assigned a given scale value, by work content scale**. Note: T = Things; D = Data; P = People; R = Reasoning; M = Math; L = Language; WI = Worker Instructions; WT = Worker Technology; SD = Worker Interaction; HEC = Human Error Consequence. Numbers in parentheses on the x axis represent the highest possible value on the scale in question; each subsequently higher scale value is represented by an increasingly darker shade of gray in each bar, (see legend for scale values associated with each shade of gray). Numbers inside bars represent the number of tasks assigned the scale value in question.

Figure 2 presents the percentage of tasks assigned a given scale value for each of the ten FJA scales described earlier. 80% of primary care tasks were rated at or below the mid-scale value across all ten scales (thus supporting hypothesis one); these tasks received low ratings on physical and interpersonal complexity (things, people), mathematical ability, autonomy (worker instructions), and worker technology; as well as low to moderate ratings on cognitive complexity (data), reasoning, language, worker interaction, and HEC.

### Objective three: differences in task complexity by function and job title

Table 4 presents analysis of variance results comparing mean differences for each scale by job title and function (means and standard deviations for each scale, by function and by job title, are available in Additional file 2). Mean ratings varied significantly by function for all scales except things and mathematics (hypothesis two; see Table 4 for F-ratios and significance values for individual scales; also, Additional file 2 denotes significant mean differences between subgroup pairs). For six of the eight scales with significant differences, service delivery and workforce management tasks exhibited significantly higher ratings than administrative duties and logistic support tasks.

**Table 4:** Analysis of variance for FJA scale ratings by job title and function

	**Source of variance**								
	Job title			Function			Job Title by function		
			
FJA scale	df	F	Sig.	df	F	Sig.	df	F	Sig.
Things	5	1.03	0.40	3	1.79	0.15	14	0.68	0.80
Data	5	3.56	0.00	3	10.20	0.00	14	0.63	0.84
People	5	3.87	0.00	3	19.34	0.00	14	1.15	0.31
Worker instructions	5	2.43	0.03	3	8.31	0.00	14	0.69	0.79
Reasoning	5	5.17	0.00	3	10.89	0.00	14	0.57	0.89
Math	5	0.83	0.53	3	1.22	0.30	14	0.30	0.99
Language	5	3.53	0.00	3	15.26	0.00	14	0.45	0.96
Worker technology	5	0.02	1.00	3	5.24	0.00	14	0.14	1.00
Worker interaction	5	0.34	0.89	3	28.41	0.00	14	0.38	0.98
Human error consequence	5	3.16	0.01	3	30.10	0.00	14	0.65	0.82

Mean ratings also varied significantly by job title for the data, people, worker instructions, reasoning, language, and HEC scales (hypothesis three). For most scales, the ratings significantly distinguished among non-adjacent job titles (with respect to training). For example, clerk and health technician tasks exhibited no significant rating differences on any of the ten scales. However, clerks and LVNs significantly differed in all six scales in which a significant effect of job title was observed, as did clerks and RNs. No significant job title by function interactions were found (hypothesis four).

**Figure 3:**
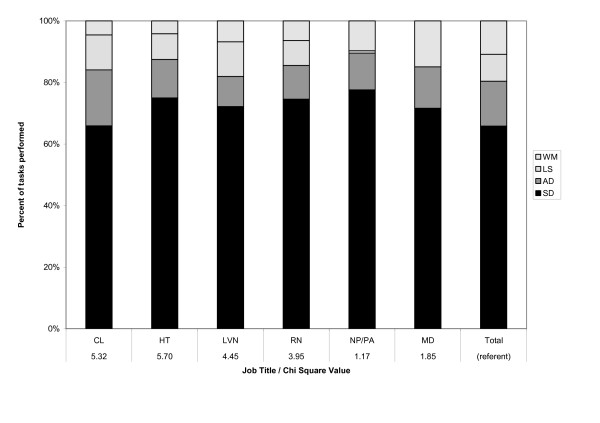
**Distribution of primary care tasks across hierarchical function, by job title**. Notes: Job title abbreviations: CL = Clerk; HT = Health Technician; LVN = Licensed Vocational Nurse; RN = Registered Nurse; NP/PA = Nurse Practitioner/Physician Assistant; MD = Physician. Each function is denoted as a shade of gray in each bar; function abbreviations (in legend): WM = Workforce management; LS = Logistic Support; AD= Administrative Duties; SD = Service Delivery. The bar labeled "total" indicates the distribution of all tasks in the task bank by function, regardless of job title. All χ^2 ^values are non significant.

This last finding was somewhat surprising. Given the differences in worker training, it was reasonable to postulate that differences in the complexity ratings of tasks performed by different job titles would be attributable to differences in the types of tasks they performed. Thus, we conducted additional analyses, comparing the distribution of tasks across the four functions for each job title. Except for physicians and nurse practitioners, who performed no logistic support tasks, all job titles performed tasks in all four functions, in proportions similar to that of the overall task bank (χ^2 ^= 1.17–5.70, n.s.); for physicians and NPs, the proportions of tasks in the remaining three functions closely mirrored the overall task bank. Figure 3 presents the proportions of tasks across functions for each job title and their corresponding chi-square values. This suggests that, rather than allocating work among the job titles by function (e.g., assigning all the administrative duties to administrative personnel and all the clinical duties to clinical personnel), all job titles were performing tasks of all kinds (including tasks that, for the higher trained personnel, did not require their level of training), thereby reducing the differences in complexity ratings of tasks by job title.

## Discussion

The present study used Functional Job Analysis to catalogue the content of work performed within primary care clinics, and explored its complexities and variations in these complexities by content and job title. Results showed that primary care is composed of four functions, the largest being service delivery; over one third of primary care tasks, however, were not directly related to patient care. This finding was also reflected in the frequency and duration data, which indicated that between 18% and 46% of primary care workers' time is spent on tasks other than service delivery, depending on the occupation. These percentages, however, should be interpreted with extreme caution, as we found high variability and unreliability in the frequency and duration data across job title and facility (see limitations section).

The first three hypotheses were supported: (1) primary care tasks rarely exceeded moderate levels of complexity along ten different dimensions; (2) service delivery and workforce management tasks were generally found to be more complex than administrative and logistical support tasks; and (3) tasks performed by personnel with more clinical training were generally more complex than tasks performed by personnel with less training. However, though this difference is statistically significant, the absolute differences were small. This might be explained in part by hypothesis four, which was not supported: job title and function did not significantly interact to impact differences in mean scale ratings. Closer examination of the data revealed that all job titles performed tasks from all four functions in similar proportions.

The study's findings are consistent with those found in the Colombia primary care study [26], which found low levels of complexity and autonomy in primary care tasks and higher complexity and HEC ratings in tasks performed by physicians versus other job titles. The Colombia study also found that registered nurses and auxiliary nurses performed almost exactly the same work (94% overlap, except for administrative duties, which were performed by the registered nurse), despite a two-year difference in training, and a 2:1 salary ratio. This, along with the complexity and autonomy findings, suggested that highly trained personnel were not being utilized to their full potential. Similar conclusions can be drawn from our findings, based both on the generally low ratings and the lack of difference across job titles in the distribution of tasks by function (hypothesis 4). Overlap analyses similar to those of the Colombia study yielded similar results; however they are beyond the scope of this report and are published elsewhere [28].

### Limitations

This study had several limitations. First, the small number of participating facilities precluded cross-facility comparisons by organizational features such as size and academic affiliation. Such comparisons may provide insights regarding the facility characteristics and/or practices that influence the allocation of work across primary care personnel. This is a clear next step in this line of research.

Second, we studied only job titles that existed at all of the sites surveyed and we explicitly excluded supervisors from the focus groups, so that SMEs would feel free to express themselves in the focus groups. These constraints may have resulted in a bias toward service delivery tasks and away from more administrative or workforce management tasks. However, managerial positions are often filled by individuals with a clinical background who participated in the focus groups appropriate to their profession. This was reflected in the resulting body of workforce management tasks. Thus, though management tasks could be under-represented in the task bank, they are certainly not absent, and provide a reminder to decision makers that workforce management must also be addressed when allocating work to primary care personnel.

Third, the task bank only examined which tasks were performed by various job titles, not how much time was spent on each task. Thus, the task bank cannot be used in its current form to make zero-sum responsibility allocation trade-offs. Although task frequency and duration data were collected, they were highly variable and unreliable, as is often the case in data of this type [46], and thus were not used in our analyses. More reliable time-use data collection methods, such as time diaries, would have placed a prohibitive burden on participants, given the number of tasks. Nevertheless, the endorsement data provide important information by identifying those tasks that have the potential for redundancy[28]. Armed with this information, decision-makers can investigate those tasks in more depth and make more evidence-based staffing and allocation decisions.

Finally, all data were collected at VA facilities, which could operate significantly differently from private or public sector primary care clinics, thereby limiting generalizability. Future studies should compare the work of primary care across these different sectors.

### Implications for science, policy, and practice

The present research, which examines the properties of tasks performed across multiple job titles in an entire health care service (primary care) for the purpose of reallocating work, is to our knowledge one of the first of its kind in American primary health care. The study contributes to science by demonstrating that a time-tested methodology for describing work performed by individual jobs can be used, with modification, to describe and make judgments about systems of work; i.e., as an evidence-based tool for health care management. Additionally, this study supports previous research demonstrating that primary care personnel are not utilized to their full potential: work functions are allocated similarly across job titles rather than tailored to the training of each job. When person-job fit is low, as when workers' tasks and training are mismatched, there is a higher likelihood of dissatisfaction; both poor person-job fit and job satisfaction have been linked to lower commitment to the organization and higher turnover intentions [7,47-51].

Underutilization of primary care personnel skills also suggests that primary care is currently more costly than it could be; the optimal staff mix of a primary care clinic could be very different from current models. Thus, there is much room to reorganize primary care work more efficiently, cost-effectively, and better matched with workers' training. We caution, however, that this redesign should not be optimized based on a single dimension, such as cost; unintended consequences (such as worker dissatisfaction and quality of care) and clinic-specific constraints (such as situations where duplication of tasks might be necessary) must be considered. Thus, individual clinics should identify their own optimal staff mix in an evidence-based manner, based on multiple factors including work characteristics (as demonstrated here), patient mix, available resources, and regulatory constraints. FJA can be used as a fact finding tool to generate important data regarding several of these factors, particularly those of fit [52]. Armed with these data and input from all relevant stakeholders, evidence-based staffing decisions are possible. Using a system like FJA to help guide these decisions is philosophically analogous to implementing evidence-based medicine to guide care quality.

## Conclusions and future directions

Given that the distribution of task functions is invariant across job titles, we conclude that primary care personnel are not utilized to the full extent of their training, despite statistically significant differences in the complexity of tasks performed by different job titles. Staffing mixes in primary care clinics should be examined to better align work and available skill, using FJA as a fact-finding tool. Future research should expand on the information collected for this study, to catalogue the specific knowledge, skills, and abilities required to perform primary care work. This information should then be used to set criteria for reaching systematic work reallocation decisions that best use employees' talents to perform the important work of primary care.

## Abbreviations used

FJA – Functional Job Analysis; HEC – Human Error Consequence; HSR&D – Health Services Research and Development Service LVN – Licensed Vocational Nurse; MD – Medical Doctor; NP – Nurse Practitioner; RN – Registered Nurse; PA – Physician Assistant; SME – Subject Matter Expert; VA – Veterans Affairs; VAMC – Veterans Affairs Medical Center

## Competing interests

The research reported here was supported by the Department of Veterans Affairs, Veterans Health Administration, Health Services Research and Development Service (HSR&D) (grant no. IIR 01-185). All three authors' salaries were supported in part by the Department of Veterans Affairs. The authors declare they have no other competing interests, financial or non-financial.

## Authors' contributions

SH was involved in all aspects of the grant that funded the work presented in this study; for this study, she interviewed participants, and was principally responsible for the design, analyses, and drafts for this manuscript. RB is the principal investigator of the grant that funded the work presented in this manuscript, and was involved in all aspects of the study, including project management, participant interviews, data analysis, and editing of manuscript drafts. FM is the senior author of the work presented in this study and of the grant that funded it – he was principally responsible for the research idea and design of the research grant that made this manuscript possible; he also participated in conducting interviews and editing drafts of this manuscript. All authors read and approved the final manuscript.

## Supplementary Material

Additional file 1 Functional job analysis protocol. Describes the steps involved in generating, editing, and ensuring the validity of a functional job analysis task bank. Click here for file

Additional file 2 Descriptive statistics for FJA scales by function, and by job title. Table presenting means and standard deviations for each of the ten FJA scales, by job title and by function. Subgroup pairs that significantly differ from each other are also noted. Click here for file
