# Supplementary material for: Are we under-utilizing the talents of primary care personnel? A job analytic examination
Source: Implement Sci. 2007 Mar 30;2:10. doi: 10.1186/1748-5908-2-10 (PMC1852323; doi:10.1186/1748-5908-2-10)
Supplement: Additional file 1 — Functional job analysis protocol. Describes the steps involved in generating, editing, and ensuring the validity of a functional job analysis task bank. [file 1748-5908-2-10-S1.doc]

Additional File 1 -- Functional Job Analysis Protocol

Generation of Tasks -- FJA Focus Groups

Two-day focus groups were conducted with the subject matter experts (SMEs) to generate a set of work outputs and tasks that are descriptive of the work that they do. Each focus group consists of 6-8 SME’s. Per traditional FJA protocol, the focus group involved facilitating participants through a series of five questions:

1. What do you get paid for? (outputs)
2. What do you need to know to do what you get paid for? (knowledges)
3. What skills/abilities do you need to apply your knowledges? (skills and abilities)
4. What do you do to get work done? (tasks)
5. What standards do you work towards, yours and your organization’s? (performance standards)

This set of questions at the end of the two days resulted in a list of detailed tasks, organized by output. Each task is written in a standard format that answers six questions:

1. Who? (performs the task -- this is always inferred to be "the worker")
2. Performs what action? (verb or verb sequence that specifically describes what is being done – worker action phrase)
3. To whom or what? (the object of the actions being performed)
4. Using what tools, equipment, work aids?
5. Using what sources of information? (knowledge, skills, abilities, instructions)
6. To produce (for achieving) what? (Expected output -- Result)

Figure 1 presents two sample tasks (one clinical, one managerial) written in standard FJA format.

Figure 1. Sample tasks in FJA format

**Task 1.1** Select non-restricted, formulary medication required by patient, type medication order in CPRS, specifying dose details (e.g., frequency, route of admission, quantity, refills, urgency, instructions), electronically sign medication order,

*using* CPRS,

*drawing* on knowledge of VA formulary and pharmacology, and

*relying* on decision-making skills, attention to detail and computer skills,

*In order to:* place formulary medication order.

**Task 2.1** Meet/discuss performance with top subordinate staff, one-on-one, reviewing with them progress made in achieving personal goals for the year, problems encountered if any (e.g., with subordinate personnel, or with equipment), training and/or self-development undertaken, innovations introduced, and goals for the coming year,

*using* documentation of recorded incidents

*drawing* on mutual experience and agency evaluation procedures

*relying* on interpersonal skills and sensitivity

*in order to*: carry out an annual performance appraisal

Ensuring the Reliability and Validity of the Task Bank

Task Bank Editing

Three certified job analysts edited tasks for grammar and to conform to the standard format prescribed by FJA. In this process, the three investigators reviewed each task and arrived at a consensus on the wording of each task. To arrive at a consensus, each task was reviewed on the basis of nine criteria, as prescribed by traditional FJA protocol[1]:

1. Does the end result of the task make a contribution to the output of which it is a part?
2. Does the language describing the worker action phrase of the task statement support the worker function levels?
3. Are the worker action phrase and the result phrase of the task statement in reasonable relation to one another?
4. Is the result identified in the task a verifiable result?
5. Can performance standards, either numerical or descriptive for the action and result, be inferred and specified?
6. Are the inferred performance standards reasonable and useful to a supervisor and to a worker?
7. Does the training content reflect the knowledges and abilities required to perform the task?
8. Is there more than a one-level spread among data, worker instructions, and reasoning scale ratings?
9. Do the verbs used in the worker action phrase of the task statement adequately express the context of the task?

If a task did not meet one or more of the aforementioned criteria, the job analysts edited the language of the task until they and the investigators all agreed that the task did meet the nine criteria. This process served as the primary means for ensuring the linguistic fidelity of the tasks.

Task Validation

The edited tasks for each job title were presented to the subject matter experts that helped generate them to ensure that after the editing they still accurately captured the work the SMEs perform. The SMEs reviewed each task statement, about which they answered each of the following two questions:

1. Does the task represent what you do (regardless of how often or how much you do it)?
2. If so, how clearly does the task describe what you actually do on the job (0 = not at all, 100 = completely clear)? The number given is called the clarity rating.

After reviewing all of the tasks, the subject matter experts were asked to rate how well (i.e., what percent) the complete list of tasks represented of their total work effort. Table A1 summarizes the results of this validation process; as can be seen from the table, subject matter experts(with the exception of health technicians) reported performing over 80 percent of the tasks presented to them, with tasks holding a mean clarity rating of about 80 percent for all job titles. This is considered in the acceptable range of reliability for functional job analysis task banks [1].

Table A1. Results of FJA task bank validations

|  |  | **Number of SMEs** | |  |  |
| --- | --- | --- | --- | --- | --- |
| **Job title** | **Facility** | *In focus group* | *Completing validation* | **Clarity*** | **% of tasks*** |
| MD | 3, 4, 6 | 14 | 7 | 86 | 95% |
| PA/NP | 1, 3, 6 | 18 | 7 | 82 | 91% |
| RN | 1, 2 | 11 | 0 | - | - |
| LVN | 1, 4, 5 | 13 | 8 | 81 | 85% |
| PSA/MAS | 2, 5 | 9 | 2 | 72 | 82% |
| Health tech | 4, 6 | 12 | 11 | 52 | 58% |
| Pharmacist | 2, 3 | 4 | 3 | 75 | 88% |

*Note: clarity refers to mean clarity rating over all tasks; percent of tasks refers to the percentage of all tasks that subject matter experts reported that they performed.

Task Ratings

The edited, validated tasks were then rated by the job analysts on each of nine dimensions (things, data, people, reasoning, mathematics, language, worker instructions (autonomy), worker technology, worker interaction, and human error consequence), as prescribed by FJA methodology. Brief scale definitions are presented in Table 2 of the main text; more detailed descriptions are documented elsewhere [1,2]. Each task was independently scaled by three raters, who then compared and reviewed their ratings to reach a consensus. Once the edited, validated tasks were assigned a consensus rating, the tasks were considered finalized, i.e., part of the permanent task bank.

References

1. Fine SA, Cronshaw SF: *Functional job analysis: A foundation for human resources management*. Mahwah, NJ: Lawrence Erlbaum Associates, 1999.

2. Fine SA, Getkate M: *Benchmark Tasks for Job Analysis: a Guide for Functional Job Analysis (FJA) Scales*. Mahwah, New Jersey: Lawrence Earlbaum Associates; 1999.
