# Supplementary material for: Are we under-utilizing the talents of primary care personnel? A job analytic examination
Source: Implement Sci. 2007 Mar 30;2:10. doi: 10.1186/1748-5908-2-10 (PMC1852323; doi:10.1186/1748-5908-2-10)
Supplement: Additional file 2 — Descriptive statistics for FJA scales by function, and by job title. Table presenting means and standard deviations for each of the ten FJA scales, by job title and by function. Subgroup pairs that significantly differ from each other are also noted. [file 1748-5908-2-10-S2.doc]

Additional file 2 -- Descriptive statistics for FJA scales by function, and by job title

|  | **FJA scale** | | | | | | | | | | | | | | | | | | | | | | | | | | | | | |
| --- | --- | --- | --- | --- | --- | --- | --- | --- | --- | --- | --- | --- | --- | --- | --- | --- | --- | --- | --- | --- | --- | --- | --- | --- | --- | --- | --- | --- | --- | --- |
|  | Things | |  | Data | |  | People | |  | WI | |  | Reas. | |  | Math | |  | Lang. | |  | WT | |  | W Int | |  | HEC | |  |
|  | M | SD |  | M | SD |  | M | SD |  | M | SD |  | M | SD |  | M | SD |  | M | SD |  | M | SD |  | M | SD |  | M | SD |  |
| *Function* | | | | | | | | | | | | | | | | | | | | | | | | | | | | | |  |
| Service delivery | 1.2 | 0.5 |  | 2.5 | 0.9 | w | 2.3 | 0.9 | w | 2.6 | 1.1 | w | 2.9 | 1.2 | w | 1.3 | 0.5 |  | 2.8 | 0.7 | w | 2 | 0.4 | w | 2.6 | 0.6 | w | 3.3 | 1.4 | w |
| Administrative duties | 1.2 | 0.4 |  | 2.1 | 1 | x | 1.5 | 0.6 | x | 2 | 0.7 | x | 2.2 | 1.1 | x | 1.2 | 0.4 |  | 2.5 | 0.8 | x | 1.8 | 0.4 | x | 2 | 0.8 | x | 2 | 0.9 | x |
| Logistic support | 1.3 | 0.5 |  | 1.5 | 0.6 | y | 1 | 0.2 | x | 1.6 | 0.5 | x | 1.4 | 0.5 | y | 1.2 | 0.4 |  | 1.9 | 0.6 | y | 2 | 0 | w,x | 1.4 | 0.6 | y | 2 | 0.6 | x |
| Workforce management | 1.1 | 0.5 |  | 2.9 | 0.7 | z | 2.3 | 1.5 | w | 2.6 | 1 | w | 3.5 | 1.2 | z | 1.1 | 0.3 |  | 3.2 | 0.6 | z | 1.9 | 0.3 | w,x | 2.4 | 1 | w | 1.8 | 1.1 | x |
|  |  |  |  |  |  |  |  |  |  |  |  |  |  |  |  |  |  |  |  |  |  |  |  |  |  |  |  |  |  |  |
| *Job title* | | | | | | | | | | | | | | | | | | | | | | | | | | | | | |  |
| Clerk | 1.1 | 0.2 |  | 1.8 | 0.7 | a | 1.6 | 0.7 | a | 1.8 | 0.6 | a | 1.8 | 0.8 | a | 1.1 | 0.2 |  | 2.3 | 0.7 | a | 2 | 0.2 |  | 2.2 | 0.9 |  | 2 | 1 | a |
| Health technician | 1.2 | 0.4 |  | 2 | 0.8 | a,b | 1.9 | 0.7 | a,b | 2 | 0.9 | a,b | 2.1 | 1.1 | a,b | 1.1 | 0.3 |  | 2.3 | 0.8 | a,b | 2 | 0.3 |  | 2.5 | 0.8 |  | 2.3 | 1.1 | a,b |
| Licensed vocational nurse | 1.2 | 0.5 |  | 2.3 | 0.9 | b,c | 2 | 0.9 | a,c | 2.3 | 0.9 | b | 2.6 | 1.2 | b,c | 1.2 | 0.4 |  | 2.6 | 0.7 | b,c | 2 | 0.3 |  | 2.4 | 0.8 |  | 2.8 | 1.3 | b |
| Registered nurse | 1.2 | 0.5 |  | 2.4 | 0.9 | c | 2.1 | 1 | b,c | 2.4 | 1 | b,c | 2.7 | 1.2 | c | 1.2 | 0.5 |  | 2.7 | 0.7 | c,d | 2 | 0.4 |  | 2.4 | 0.8 |  | 2.9 | 1.4 | b,c |
| Nurse pract/ phys. Asst. | 1.2 | 0.5 |  | 2.8 | 0.9 | d | 2.3 | 1.1 | b,d | 2.7 | 1.1 | c,d | 3.2 | 1.2 | d | 1.3 | 0.5 |  | 2.9 | 0.7 | d,e | 2 | 0.5 |  | 2.5 | 0.8 |  | 3.3 | 1.6 | c |
| Physician | 1.2 | 0.5 |  | 2.8 | 0.9 | d | 2.4 | 1.1 | b,d | 2.7 | 1.1 | d | 3.2 | 1.2 | d | 1.3 | 0.5 |  | 3 | 0.7 | e | 2 | 0.4 |  | 2.5 | 0.7 |  | 3.2 | 1.6 | c |

Notes: WI = Worker Instructions; Reas. = Reasoning; Lang.= Language; WT = Worker Technology; W Int = Worker Instructions; HEC = Human Error Consequence. For each scale, cell pairs within function and within job title with common superscripts are not significantly different (e.g., service delivery and workforce management tasks are not significantly different on the people scale; similarly, clerk tasks and health technician tasks are also not significantly different on the people scale.
